# Feeding Canola, Camelina, and Carinata Meals to Ruminants

**DOI:** 10.3390/ani9100704

**Published:** 2019-09-20

**Authors:** Eduardo Marostegan Paula, Lorrayny Galoro da Silva, Virginia Lucia Neves Brandao, Xiaoxia Dai, Antonio Pinheiro Faciola

**Affiliations:** 1 Instituto de Zootecnia, Centro APTA Bovinos de Corte, Sertãozinho SP. 14174-000, Brazil; emarostegandepaula@gmail.com (E.M.P.); lorraynyg@hotmail.com (L.G.d.S.); 2 Department of Animal Sciences, University of Florida, Gainesville, FL 32608, USA; v.nevesbrandao@ufl.edu (V.L.N.B.); x.dai@ufl.edu (X.D.)

**Keywords:** animal growth, animal performance, digestibility, milk production, mustard, oilseed, ruminal fermentation

## Abstract

**Simple Summary:**

The world population is estimated to reach 9 billion people by 2050, which is estimated to increase the demand for food, fuel, and fiber by 60%. Domesticated ruminants play a vital role in this scenario because they can consume food byproducts that are nonedible for humans, contributing to livestock sustainability. Meals extracted from oilseed plants, such as soybean, canola, carinata, and camelina, are examples of food byproducts. Soybean meal is likely the byproduct most used worldwide, due to its availability and high-quality nutritional composition. However, the dependency on monocultures such as soybean is problematic due to price fluctuation and, in some countries, import dependency. Canola, camelina, and carinata meals have been investigated in the past two decades. Therefore, we aimed to summarize the results from studies in which canola, camelina, and carinata meal were fed to ruminants in order to evaluate how comparable these are to soybean meal and other common protein supplements in terms of animal digestion and performance. Based on this review, we conclude that canola meal is at least as good as soybean meal; and that camelina and carinata meal can be a valuable alternative feedstuff for livestock animals.

**Abstract:**

Soybean meal (SBM) is a byproduct from the oil-industry widely used as protein supplement to ruminants worldwide due to its nutritional composition, high protein concentration, and availability. However, the dependency on monocultures such as SBM is problematic due to price fluctuation, availability and, in some countries, import dependency. In this context, oilseeds from the mustard family such as rapeseed/canola (*Brassica napus* and *Brassica campestris*), camelina (*Camelina sativa*), and carinata (*Brassica carinata*) have arisen as possible alternative protein supplements for ruminants. Therefore, the objective of this comprehensive review was to summarize results from studies in which canola meal (CM), camelina meal (CMM), and carinata meal (CRM) were fed to ruminants. This review was based on published peer-reviewed articles that were obtained based on key words that included the oilseed plant in question and words such as “ruminal fermentation and metabolism, animal performance, growth, and digestion”. Byproducts from oil and biofuel industries such as CM, CMM, and CRM have been evaluated as alternative protein supplements to ruminants in the past two decades. Among the three plants reviewed herein, CM has been the most studied and results have shown an overall improvement in nitrogen utilization when animals were fed CM. Camelina meal has a comparable amino acids (AA) profile and crude protein (CP) concentration to CM. It has been reported that by replacing other protein supplements with CMM in ruminant diets, similar milk and protein yields, and average daily gain have been observed. Carinata meal has protein digestibility similar to SBM and its CP is highly degraded in the rumen. Overall, we can conclude that CM is at least as good as SBM as a protein supplement; and although studies evaluating the use of CMN and CRM for ruminants are scarce, it has been demonstrated that both oilseeds may be valuable feedstuff for livestock animals. Despite the presence of erucic acid and glucosinolates in rapeseed, no negative effect on animal performance was observed when feeding CM up to 20% and feeding CMN and CRM up to 10% of the total diet.

## 1. Introduction

The global population is estimated to reach 9 billion people by 2050, leading to a 60% increase in the demands for food, fuel, and fiber [1]. In this scenario, ruminant animals, such as cattle and sheep play an important role in livestock sustainability [2]. These animals are able to consume crop residues and food byproducts that are nonedible for human consumption and that otherwise would become an environmental issue [3]. Soybean meal (SBM) is a byproduct from the oil-industry widely used as protein supplement to ruminants worldwide due to its nutritional composition. This byproduct is known to have high protein concentration and quality and is widely available. However, the dependency on monocultures such as SBM is problematic due to price fluctuation, availability and, in some countries, import dependency. In this context, oilseeds from the mustard family (Brassicaceae) such as rapeseed/canola, camelina, and carinata have arisen as possible alternative protein supplements to be used in ruminants’ diets.

Rapeseed (*Brassica napus* and *Brassica campestris*) is a bright-yellow flowering plant cultivated worldwide due to the oil concentration of its seed. However, rapeseed may contain high levels of erucic acid and glucosinolates, which are antinutritional factors that may compromise animal performance [4]. Canola, on the other hand, is an offspring of rapeseed (*Brassica napus* and *Brassica campestris/rapa*) that was bred through standard plant breeding techniques to have low levels of erucic acid (<2%) in the oil portion and low levels of glucosinolates (<30 µmol/g) in the meal portion [4]. In Europe and other countries, the terms “double-zero rapeseed” and “double low” (low erucic acid and low glucosinolates) are used to refer to “canola quality” seed, oil, and meal [4]. As mentioned before, erucic acid and glucosinolates are antinutritional factors toxic to animals which may affect digestion and health of most animals [5,6], and consequently limit rapeseed meal inclusion levels in animal diets to very low amounts. Therefore, this comprehensive review is focused on studies that reported the use of canola meal (CM) and double-zero rapeseed meal on ruminants’ diets.

Camelina (*Camelina sativa*) is an oilseed plant used for biofuel production. Compared to other convectional oilseeds, camelina combines agronomic features such as low-nutrient requirements and good resistance to disease, pests, and drought, which makes it well-adapted to low-input farming [7,8,9]. Camelina meal (CMM) is the byproduct of camelina seed that contains lipids with significant amounts of the essential fatty acids 18:2n-6 and 18:3n-3. It is also relatively rich in crude protein (CP) concentration and essential amino acid (AA), indicating that it could be a potential alternative protein and energy source for ruminants. However, glucosinolates and erucic acid present in camelina byproducts could impair DMI and thyroid function in animals [5,10].

Carinata (*Brassica carinata*), also known as Ethiopian mustard, is also an oil seed plant largely used for biofuel production [11] and as leaf vegetable in some parts of Africa [12]. Carinata meal (CRM) has approximately 50% crude protein (CP, 17% neutral detergent fiber (NDF), and 10% acid detergent fiber (ADF; dry matter, DM, basis). Additionally, CRM has approximately 8% neutral detergent insoluble CP (NDICP) and 1.4% acid detergent insoluble CP (ADICP) as % of CP [11,13]. In terms of fatty acid profile, carinata seed has high concentration of unsaturated fatty acids such as 18:2n-6 and 18:3n-3 [13,14]. Similarly to other plants from the Brassicaceae family, carinata also contains antinutritional factors such as glucosinolates (115.2 µmol/g in the meal) [14] and erucic acid (40% of total fatty acids) [12]. However, genetic breeding and manipulation has been able to develop zero erucic carinata, which contains 5 to 10% of the total fatty acid as erucic acid [15]. Compared to CM, CRM has greater CP concentration, lower fiber and rumen undegraded protein (RUP) [16] concentration, and similar fermentation parameters and metabolizable protein supply.

Therefore, the overall objective of this comprehensive review was to summarize the results from studies in which CM, CMM, and CRM were fed to ruminants and their effects on dry matter intake (DMI), ruminal fermentation, microbial population, digestion, metabolism, milk production and composition, and animal growth. Therefore, this review provides an overview of the utilization of CM, CMM, and CRM as protein supplements for ruminants.

## 2. Chemical Composition

### 2.1. Canola Meal

Typical CM chemical composition has been reported by [17] and several studies [17,18,19,20,21,22,23,24,25,26,27,28,29,30,31,32,33,34,35,36,37,38,39,40,41,42,43,44,45] (Table 1; Table 2). Canola meal has approximately 91% DM and 92% organic matter (OM, % of DM). The ether extract (EE) concentration ranges from 2.3 to 7.4% (DM basis) depending upon the oil extraction method (Table 1). Compared with other oilseed meals, CM has been reported to be a good source of essential minerals. The meal has approximately 8% ash (% of DM) and its mineral content is composed of 0.9% Ca, 1.1% P, 0.6% Mg, 1.1% K, 0.23% Na, and 0.11% Cl (% of DM; Table 1). Carbohydrate and fiber contents of CM is composed of approximately 29% NDF, 20% ADF, 20% non-fiber carbohydrate (NFC), and 4% starch (% of DM) with a greater NDF and ADF concentration compared to SBM [40]. The lignin concentration of CM is approximately 10% [32,44].

Canola meal is described as having approximately 40% CP, 18% neutral detergent insoluble nitrogen (NDIN), and 5% acid detergent insoluble nitrogen (ADIN; % of N), and 57% rumen degraded protein (RDP) and 43% RUP (% of CP). In the last decade, CM availability has increased due to production increase [46]. In addition, CM has been widely used as a protein supplement for ruminants [47]. Overall, CM has lower CP and greater fiber concentration than SBM [40]; however, studies have suggested that CM CP is used more efficiently than SBM CP by lactating dairy cows [41,47]; these results will be discussed in detail in Section 6. Huhtanen et al. [48] demonstrated in a meta-analysis that RUP and metabolizable protein (MP) concentrations in CM were similar to those from SBM. However, a greater concentration of RUP in diets containing CM compared to SBM was reported by Brito et al. [49]. Approximately 40% of the total AA present in CM are essential AA, CM has approximately 2.52% histidine (His), 4.87% lysine (Lys), 1.88% methionine (Met), and 5.90% arginine (Arg) [22,39,50]. Moreover, NRC [51] reported a greater Met concentration in CM than in SBM.

As previously mentioned, canola, an offspring of rapeseed (*Brassica napus* and *Brassica campestris/rapa*), was bred through standard plant breeding techniques with the aim to reduce the levels of the antinutritional factors such as erucic acid (<2%) in the oil portion and glucosinolates (<30 µmol/g) in the meal portion [32]. Total glucosinolates concentration in traditional rapeseed meal is approximately 50–100 μmol [32]. In CM it varies according to the processing method; however, CM has been reported to contain approximately 2 μmol/g [37,50]. High levels of glucosinolates are known to negatively affect thyroid function by inhibiting thyroid hormone production and to reduce DMI due to its bitter taste [52]. However, in a meta-analysis study, Martineau et al. [53] reported greater DMI and milk yield in dairy cows fed CM (or a low-glucosinolate rapeseed meal) compared to several other commonly fed protein supplements.

### 2.2. Camelina Meal

Camelina meal contains lipids with significant amounts of essential fatty acids 18:2n-6, 18:3n-3 (Table 3). The average EE concentration in CMM is 5.76% (DM). The CMM has variable amount of residual oil (5 to 26% DM) depending on oil extraction efficiency [54]. However, it is also relatively rich in CP and essential AA (Table 3 and Table 4). The average CP concentration of CMM is 45.7%, (DM basis).

The AA profile of CMM is similar to CM [26]. The feasibility of the use of alternative grain legumes in ruminant diets is determined not only by their chemical composition, but also by the rate and extent of degradation of nutrients in the rumen. Ruminal degradability of camelina protein in situ (76%) was greater than that of soybean (58%) or rapeseed (52%; [10]), limiting their production responses in high-yielding ruminants. Meanwhile, CMM contains significant amounts of fiber, 37.0% on average in DM bases (Table 3), and various antinutritional factors, e.g., glucosinolates (Table 3), sinapine, and erucic acid (Table 5), that limit their use in animal feeds [54,62,63].

### 2.3. Carinata Meal

It has been reported that in carinata seed, erucic acid makes up 40% of the total fatty acids [12]. However, genetic breeding and manipulation could potentially reduce this concentration to close to zero. In a study crossing *B. carinata* with *B. napus* containing low erucic acid, a zero erucic carinata was developed [15]. Additionally, the studies of Velasco et al. [15] successfully isolated low erucic acids mutants, ranging from 5 to 10% in total fatty acid. Interestingly, the low erucic acid plants also had significantly greater oleic acid concentration [12].

Carbohydrates concentration in carinata seed has been described [11], in which NDF ranged from 9.67 to 13.4%, ADF from 4.95 to 6.43%, and NFC from 66.4 to 68.1%, differing according to cultivar. CP has been reported to range from 23.1 to 24.8% in different cultivars [60]. Carinata seed is high in unsaturated fatty acids and the fatty acid profile is presented in Table 6.

Currently, there are governmental policies that stimulate biofuel production, especially in North America [67], which has increased the production of byproducts. Consequently, the use of alternative feedstuff for cattle nutrition has increased, primarily for dairy replacement heifers and beef animals [68]. Carinata meal (CRM) has approximately 48.2 to 53% CP, 14.4 to 18.8% NDF, 10.2 to 11.4% ADF as % DM, 6.0 to 9.54% NDICP, and 1.26 to 1.6% ADICP as % CP [12,66]. Overall, CRM has greater CP and lower fiber concentrations than CM. Similar to other plants from the Brassica family, carinata also has antinutritional factors such as glucosinolates. Ban et al. [14] reported a glucosinolate concentration of 115.2 µmol/g in the meal, and 168.5 µmol/g in the pressed cake. An overall chemical composition of carinata seed, pressed cake, and cold-pressed meal is presented in Table 7.

In a study using in situ methodology to estimate ruminal degradation and a three-step in vitro procedure [70] to estimate intestinal degradation, Xin and Yu [60] reported that CRM had lower RUP concentration and similar fermentation parameters and metabolizable protein supply compared to CM. Similarly, in a study using the same methods [16], Ban et al. [14] reported extensive ruminal degradation, with lower RUP in CRM compared to CM, possibly due to its greater soluble protein.

Ruminal degradation and intestinal digestibility of CRM were determined by Lawrence and Anderson [71]. This study aimed to compare ruminal degradation and intestinal digestibility of CRM to multiple protein sources, such as camelina, CM, SBM, linseed meal, and dried distillers grains with solubles (DDGS). For that, these authors used solvent-extracted CRM, CM, linseed, SBM, and DDGS, while camelina was obtained through cold-press extraction. Therefore, the EE of each protein supplement was different and ranged from 1.8% (SBM) to 14.3% (CRM). It was reported that CRM had the greatest ruminal DM disappearance and the lowest potentially degradable DM fraction. Similarly, when CP was evaluated, CRM had the greatest A fraction and least C fraction. In this study, CRM had a total digestible CP comparable with camelina, soybean, and linseed, of approximately 95%. Camelina and CRM had the greatest RDP and least RUP. However, when intestinal CP digestibility was evaluated, using the three-step procedure [72], soybean meal had the greatest values, and CRM the lowest.

## 3. Dietary Inclusion and Effects on Dry Matter Intake

### 3.1. Canola Meal

As mentioned earlier, the concentration of glucosinolates in the meal portion of rapeseed can be a concern due to its negative effects on animal health, and consequently limit its inclusion levels in the diet. Canola meal has been extensively studied in feeding trials with lactating dairy, and the results on DMI reported in the literature have been inconsistent when compared to other common protein supplements. Broderick et al. [40] evaluated the inclusion of CM in the diet at two levels (11 and 17%) of DM, and the authors observed an increase in DMI of 0.4 kg/d for CM in both levels compared to SBM in isonitrogenous diets. Rinne et al. [71] compared the inclusion of CM in the diet at high (15.6% of DM) and low (8.4% of DM) levels compared to SBM at high (12.4% of DM) and low (6.7% of DM) levels. Contrary to Broderick et al. [40] they did not observe differences among dietary treatments. Brito and Broderick 45] evaluated the DMI of lactating dairy cows supplemented with urea, cottonseed meal (CSM), SBM, or CM in isonitrogenous diets. The authors observed a significant increase in DMI for CM compared to SBM diets and no differences were observed when compared to CSM, 24.9, 24.2, and 24.7 kg/d, respectively. In this trial, the inclusion of CM in the diet was at 16.1% of DM.

Mulrooney et al. [19] conducted a study evaluating the effects of CM replacing DDGS in different proportions; the dietary treatments were 100% CM (6.63% of DM), 66% CM (4,59% of DM), 33% CM (2.29% of DM), and 0%. They observed no differences in DMI. Contrarily, Chibisa et al. [73], evaluating the effects of replacing CM with increased levels of wheat-DDGS (0, 10, 15, and 20% of DM) and observed an linear increase in DMI (1.1 kg/d, on average) for diets with wheat-DDGS when compared to CM diet. Maesoomi et al. [18], evaluating CM as an alternative supplement to CSM in the diet of midlactation dairy cows, did not observe differences in DMI among dietary treatments, which were: 100% CM (14.3% of DM), 50% CM 50% CSM (9% of DM), and 100% CSM (no CM added in the diet). Christen et al. [30] evaluated four protein supplements, SBM, high protein dried distillers grains (HPDDG), CM, and DDGS, in isonitrogenous diets on DMI in dairy cows and they did not observe differences in DMI among treatments. Similarly, Swanepoel et al. [34] did not observe a DMI effect when evaluating the combination of CM and HPDDG with solubles from corn in dairy diets of early lactation high producing dairy cows. The inclusion rate of both supplements (DM basis) were as follows: 1) 0% CM + 20.2% HPDDG; 2) 6.61% CM + 13.6% HPDDG; 3) 13.5% CM + 6.79% HPDDG; 19.9% CM + 0% HPDDG. Acharya et al. [31] also observed no differences in DMI for cows fed diets with CM or HPDDG with two levels of CP in the diet—14 and 16% CP (DM basis). The dietary inclusions of CM in this trial were 9.23% and 15.9% of DM, respectively. Martineau et al. [53] conducted a meta-analysis where they evaluated the effects of CM compared to other protein supplements on DMI. They reported an increase of 0.24 kg/d on average for cows fed CM at a 10% inclusion rate (DM basis).

Overall, CM has been included from 6 to 20% of total DM without comprising animal performance and, in some cases, enhancing the DMI of lactating dairy cows. Furthermore, to the best of our knowledge, varieties of canola or double zero rapeseed, when added to the diet of dairy cows, have not been reported to affect animal health.

### 3.2. Camelina Meal

The glucosinolates concentration in CMM is 23.1 µmol/g on average across different genotypes [59]. Camelina products are considered of moderate toxicity with regards to their glucosinolate concentration [62], but it still has potential to affect thyroid gland function and cause metabolism disturbances [54]. Therefore, the American Food and Drug Administration restricted the inclusion level for camelina products in beef cattle feedlot rations to a maximum of 10% of dietary DM. Camelina oil contains 2–5% erucic acid (22:1n9; 7), as does camelina seed (Table 3). Erucic acid was found to induce myocardial lipidosis in rats and was regulated to a maximum of 2% (USA) and 5% (EU) of the fat in food products [5,74].

Feeding unprocessed or processed camelina seeds to ruminants has sometimes, but not always, decreased DMI, which may be related to glucosinolate concentration [10]. Supplemental CMM has been reported to reduce DMI compared to feeding camelina oil [75] or whole seeds [64]. Intake of diets based on red cover silage has been shown to be unaffected or marginally decreased in response to plant oil (rapeseed, sunflower seed, camelina seed, or linseed) supplements [75,76]. However, consuming 2.04 kg of DM/d of CMM has been shown to impair DMI in beef cattle when compared to SBM [65]. However, no effects on DMI were observed when CMM was included at 10% of the diets for dairy heifers [10], as well as at 0.95 kg/d in the diet of beef heifers [56] when compared to linseed meal and SBM, respectively. Reductions in DMI associated with lipid/oilseed byproduct consumption have often been attributed to the negative effects of unsaturated fatty acids on ruminal OM degradation, ruminal fermentation, and the tendency to shift the site of digestion from the rumen to the intestine [77]. Therefore, the reduction in DMI by CMM supplements also may be related to the polyunsaturated fatty acids (PUFA) concentration in CMM. Conflicting results may be due to different CMM inclusion levels, different animal physiological stages, and different basal diets used.

### 3.3. Carinata Meal

The Association of American Feed Control Officials (AAFCO) recommends that meals containing glucosinolates should not be fed at more than 10% DM [74]; therefore, the inclusion level of CRM follows the same restriction. Studies using CRM for dairy or beef cattle are still scarce, and to our knowledge there are only three published studies [68,78,79]. Rodriguez-Hernandez and Anderson [68] determined the effects of feeding CRM to dairy heifers on growth, ruminal fermentation, and nutrient utilization. In that study, 24 Holstein heifers were fed 10% cold-pressed CRM (% DM) for 11 months. In order to have isonitrogenous and isoenergetic diets, and due to relatively high EE concentration in CRM (20.1%, DM), animals were fed a control diet containing dried distillers grains with solubles (DDGS, 9.0%, DM). Carinata had 38% CP and 45.9 µmol/g of glucosinolates. In this study, it was reported no differences in DMI, BW, ADG, feed efficiency and frame size between heifers fed CRM or control.

In an experiment carried out over two consecutive years [78], eight steers were used in a duplicated 4 × 4 Latin square and fed 1.39 kg/d of CRM (approximately 0.3% BW). Authors reported that there were no effects on DMI for steers fed CRM compared to steers fed either CSM, DDGS, or SBM. It is has been hypothesized that ruminants are less sensitive to the deleterious effects of feeding meals containing glucosinolates, and in fact, when increasing levels of CRM (from 0 to 35%, % of DM) were added in the diet of broilers, a linear reduction in DMI was observed [80], suggesting that ruminants may be more resistant to the negative effects of glucosinolates.

## 4. Effects on Ruminal Fermentation and Microbial Population

### 4.1. Canola Meal

Broderick et al. [40] did not observe a difference between CM and SBM diets in terms of total VFA, acetate, propionate, butyrate concentration, or acetate:propionate ratio. However, they observed lower NH_3_-N and total branched-chain VFA (BCFVA) concentrations for cows fed CM diets compared to SBM. Similarly, Paula et al. [24] did not observe differences in total and individual VFA and NH_3_-N concentration, but they did observe a reduction in total BCVFA when diets with CM were fed compared to SBM using an in vitro dual flow continuous culture system. On the contrary, Brito et al. [49] did not observe differences in total and individual VFA and NH_3_-N concentrations and BCVFA among diets supplemented with CM, SBM, or CSM fed to dairy cows. In addition, other studies evaluating ruminal fermentation parameters in dairy cows fed diets supplements with CM vs. SBM did not observe differences in any VFA traits [25,75]. Comparing CM vs. dried distillers grains, Mulrooney et al. [19] did not observe differences in total and individual VFA concentration, except for valerate, which linearly increased when CM was gradually replaced with DDGS. Similarly, Chibisa et al. [73], evaluating the effects of replacing CM with increased levels of wheat-DDGS (0, 10, 15, and 20% of DM), did not observe difference for all VFA traits, except isobutyrate was greater for CM compared to diets with 20% wheat-DDGS. Christen et al. [30] did not observe differences in any VFA traits and NH_3_-N concentration among four protein supplements, SBM, HPDDG, CM or DDGS, in isonitrogenous diets fed to dairy cows. Overall, the effects of CM vs. other protein supplements on total VFA, acetate, butyrate, and propionate concentrations have been minor. Most of the effects observed by the studies cited herein were related with ruminal metabolites (e.g., BCVFA) related to protein degradation pathways.

### 4.2. Camelina Meal

Among dietary components, such as carbohydrate, protein, and lipids, ruminal microorganisms are most sensitive to the amount and composition of lipids, especially the ones high in PUFA. Camelina products, which are high in lipids, typically contain significant amounts of essential fatty acids 18:2n-6 and 18:3n-3, thus one would expect them to have effects on the ruminal environment and microbial population. Hurtaud and Peyraud [64] found that intakes of CMM at 2 kg/d decreased molar proportion of acetate and increased the molar proportion of propionate and valerate compared to SBM in Holstein dairy cows. Brandao et al. [55] also found that replacing 50% or 100% of CM with CMM (solvent-extracted) linearly decreased the molar proportion of acetate and increased the molar proportion of propionate in a dual-flow continuous culture system developed to mimic in vivo ruminal microbial fermentation. Similar results were also found with intakes of 630 g/d of camelina seed for lactating cows [64] and when it was fed at 7.7% and 17.7% in the diet in dual-flow continuous culture system [26].

Dietary PUFA typically modify ruminal fermentation, characterized by a shift towards propionate at the expenses of acetate, butyrate, or both lipogenic VFA [64,81,82], which may be related to the toxic effects of linoleic acid and alpha-linolenic acid on specific cellulolytic and butyrate-producing bacteria [83,84]. Increase in propionate concentration and reduction in acetate by CMM supplements suggest that CMM supplements could confer energetic benefits to ruminants.

However, feeding CMM at 10% of the diet to growing dairy heifers did not affect ruminal fermentation parameters [10]. Dietary inclusion of camelina oil up to 6% also did not have a major influence on ruminal fermentation in lactating cows [85,86]. The lack of effects of camelina on ruminal fermentation may be related to the lack of effects of camelina on ruminal bacteria, fungi, and protozoa in these studies [85,86]. None of the reported studies that tested CMM observed effects on total VFA concentration. Therefore, CMM appears to have no effects on overall microbial fermentation but may alter ruminal bacterial community composition and therefore change ruminal fermentation end products’ molar proportions.

Dai et al. [87] found that supplementation of camelina seed changed ruminal bacterial community composition in both the liquid and solid fractions; the main cellulolytic bacteria were decreased while propionate-producing bacteria were increased by camelina seed in the dual-flow continuous system, which could explain the reduction in acetate molar proportion and increased propionate molar proportion by camelina seed in these studies. The inconsistent results on ruminal fermentation end products by CMM supplementation may be due to the differences in protein sources, the forage:concentrate ratio, and basal diet composition.

### 4.3. Carinata Meal

The study of Schulmeister et al. [78] was performed using solvent-extracted pellets of CRM. This meal contained 43.3% CP and 28 µmol/g glucosinolates. The authors reported that feeding CRM did not affect ruminal pH, NH_3_-N concentration, or total VFA concentration. It was observed that steers fed SBM had a greater molar proportion of BCVFA than animals fed CRM. Similarly, the study of Rodriguez-Hernandez et al. [68] also reported no effects on ruminal pH, NH_3_-N, and total VFA concentration when CRM was fed to replacement dairy heifers, compared with heifers fed dried distillers grains. However, they observed that feeding CRM increased the molar proportions of acetate and isovalerate. Interestingly, even though the study of Schulmeister et al. [78] used solvent-extracted CRM (hexane), and Rodriguez-Hernandez et al. [68] used cold-pressed CRM, both studies observed similar responses of ruminal fermentation.

## 5. Effects on Digestion and Metabolism

### 5.1. Canola Meal

Studies in the literature have reported a mix of responses for total tract digestibility when SBM was replaced by CM in the diet of dairy cows. Brito and Broderick [44] compared the effects of CM and SBM of cows fed alfalfa and corn silages (20.7% of alfalfa silage and 35.0% of corn silage, DM basis), and observed greater NDF digestibility for cows fed CM diets compared to SBM, but no difference for other nutrient digestibilities. Paula et al. [25] observed greater total tract digestibility for cows fed CM diets compared to SBM of when cows were fed 30% alfalfa silage and 30% corn silage (DM basis). We have evaluated total tract digestibility of cows fed diets with three different proportions of alfalfa and corn silages (50:10, 30:30, and 10:50; DM basis) with either SBM or CM as protein supplements [88], and we have observed that when alfalfa silage was fed in greater proportions in the diet, DM, OM, CP, and NDF apparent total tract digestibility for diets supplemented with SBM was greater compared to CM. However, when corn silage was fed in greater proportions in the diet, CP digestibility was greater for CM diet compared to SBM. No differences between protein supplements were observed for cows fed 30:30 alfalfa to corn silage ratio (DM basis). Recently, Sánchez-Duarte et al. [89] observed greater DM, OM, NDF, and ADF apparent total tract digestibility for diets supplemented with SBM vs. CM, regardless of the starch inclusion level in the diet (21 and 27% DM). Maesoomi et al. [18] observed greater DM and CP apparent total tract digestibility for diets supplemented with CM compared to cottonseed meal in the diets of mid-lactation Holstein cows. In an meta-analysis with 43 published peer-reviewed studies with dairy cows that were fed grass silage or with partly replaced grass silage with legume or whole-crop cereal silage, Huhtanen et al. [48] evaluated CM as a protein supplement vs. SBM on the estimated true total tract digestibility of CP, and the authors did not observe differences between the two supplements. Overall, the differences in total tract digestibility between CM and SBM diets were due to the minor differences in ingredient and chemical composition concentration in the diets, since diets supplemented with CM usually have greater NDF and lower NFC concentrations in isonitrogenous diets compared to SBM. However, these differences do not seem to affect cows’ performance and the performance of cows fed CM, which is likely related to the AA profile of CM.

Mustafa et al. [28] evaluated the effects of feeding high fiber and regular CM vs. SBM on blood urea N and they reported a reduction of 13% for cows fed both CM compared to SBM. Swanepoel et al. [34] reported a linear increase for all essential AA, except His, in plasma with increasing levels of CM in the diet replacing HPDDG. They also reported that the plasma Lys to Met ratio of 3:1 recommended by NRC [51] was reached when cows were fed between 6.5 and 13.5% of CM and 13.6 and 6.79% of HPDDG (DM basis). Similarly, Acharya et al. [31] observed greater concentration of all essential AA, except Met and His, for lactating cows fed diets with CM compared to diets with HPDDG, regardless of dietary CP. Furthermore, they observed that Met was the first limiting AA for cows fed CM diets, whereas Lys was the first limiting AA for cows fed HPDDG diets. Maxin et al. [39] observed the highest plasma essential AA, except for Leu, when CM diets were fed compared to HPDDG, and greater Met concentration when CM was fed compared to SBM and no differences among CM and HPDDG and wheat DDGS. No differences in Lys concentration were observed among diets. Furthermore, the author did not observe differences in total essential AA. Contrarily, Christen et al. [30], comparing diets with HPDDG vs. SBM, CM, and DDGS, observed a greater concentration of total essential AA in plasma for HPDDG and SBM, intermediate values for DDGS, and the lowest concentrations when CM diets were fed. In terms of limiting AA, they observed that Met was the first limiting AA when SBM diets were fed and Lys was the first limiting for the other three diets, and Rinne et al. [71] did not observe differences for individual and total essential AA concentrations in plasma of dairy cows fed red clover and grass silage diets with CM or SBM as protein supplements. Most of the studies cited herein suggest that dairy cows fed CM as protein supplement increased the concentration and total essential AA in plasma, and these results are in line with the meta-analysis study of Martineau et al. [90] where they reported an increase in total essential AA, branched-chain AA, and all individual AA in plasma and a reduction in blood urea N when dairy cows were fed CM vs. other protein supplements.

### 5.2. Camelina Meal

Inclusion of CMM had no effects on total tract digestibility in lactating cows (29 g/kg of CMM) [75] when fed to steers at 2 kg of DM/d [65], and to dairy heifers as 10% of the diet [10]. Meanwhile, inclusion of camelina oil up to 6% (DM) in the diet also did not affect total tract digestibility in lactating cows [85,86]. Plant oils, oilseeds, and their byproducts have variable effects on diet digestibility depending on the concentration and source of added lipids and the composition of the basal diets, in which digestibility reductions are often attributed to the negative effects of unsaturated fatty acids on the ruminal microbial population [91,92]. Camelina meal contains a significant amount of essential fatty acids 18:2n-6, 18:3n-3; however, the average EE concentration in CMM is relatively low (5.76% in DM), and thus the amount of unsaturated fatty acid that reaches the rumen is typically low and thus has minor effects on ruminal bacteria, especially those involved in fiber digestion or biohydrogenation, which could explain the lack of effects of CMM on ruminal fermentation [55,57]. Brandao et al. [55] observed a linear reduction in NDF degradation in dual-flow continuous culture system, which could be due to the greater inclusion of CMM (10.1 to 20.2% in DM) in the diet than the levels recommended by the FDA (≤10%). These suggest that CMM can replace conventional protein and energy supplements in the diets of ruminants.

Glucosinolates are polar compounds present in camelina and they have the potential to lower thyroid gland function and cause metabolism disturbances [63]. Thyroid hormones are essential for the control of basal metabolic rate and normal development of animals. Mawson et al. [6] reported that glucosinolates reduced the circulating concentration of triiodothyronine (T3) and thyroxine (T4) and may overstimulate thyrotropin-stimulating hormone secretion, causing hypertrophy of thyroid tissues. Lardy and Kerley [63] reported that beef steers supplemented with rapeseed meal with glucosinolates had decreased serum concentration of T4. However, feeding CMM at 2 kg DM/d to steers did not alter thyroid gland function [65]. Supplemental CMM (0.95 kg/heifer/d) for beef heifers also did not affect the serum concentration of T4 [56]. Furthermore, 10% dietary inclusion of CMM did not affect T3 and T4 concentration in beef heifers [10]. The concentration of glucosinolates in CMM is relatively low (23.1 µmol/g) compared with conventional rapeseed meal (90 to 140 µmol/g [63]), which may explain the lack of detrimental effects of CMM on thyroid function. Therefore, effects of glucosinolates on thyroid function depends on the dietary inclusion level of CMM.

Meanwhile, inclusion of CMM had no effects on plasma nonester fatty acids and glucose concentration [11,56,78]. However, infusion of CMM significantly increased the concentration of PUFA (C18:2 and C18:3) and decreased the concentration of saturated and monounsaturated fatty acids in the plasma of dairy cows (C16:1 and C18:1; 30), and increased the circulating concentration of PUFA in beef cattle when 2 kg DM/d of CMM was fed [65], which could be due to the increase of PUFA in the diets supplemented with CMM.

### 5.3. Carinata Meal

The effect of cold-press CRM on total tract digestibility was evaluated by [68,82]. Feeding CRM decreased digestibility of DM, NDF, and ADF and did not change CP and OM digestibility when compared with heifers fed dried distillers grains [68]. Even though they observed differences in nutrient digestibility, the ADG was not affected by treatment, demonstrating that these differences in digestibility were not sufficient to result in deleterious effects on animal performance. When solvent-extracted CRM was fed to steers, there was no effect on digestibility of DM, OM, CP, NDF, and ADF. It can be speculated that the negative effects of CRM on nutrient digestibility observed in a previous study [68] is likely due to greater EE concentration, which can depress digestibility. However, feeding CRM seems to not affect animal performance, based on ADG reported by [68,78,79].

In the study of [78], blood samples were collected immediately before feeding, and every three hours until a cycle of 24 h was complete. They did not observe a treatment effect on blood glucose concentration or plasma urea nitrogen. Due do possible deleterious effects of antinutritional CRM factors on thyroid hormones and reproduction, [79] blood samples were collected every seven days for 70 days for later analysis of thyroid hormones and blood metabolites. They used 64 beef heifers, fed 0.3% BW of pelleted solvent-extracted CRM (2.5% EE), and reported no difference on triiodothyronine and thyroxine hormones concentration, as well as haptoglobin. However, ceruloplasmin concentration decreased in animals fed CRM compared with heifers fed a control diet containing only bermudagrass. Additionally, these authors evaluated time to attainment of puberty and concluded that CRM did not affect it.

## 6. Effects on Milk Production and Composition

### 6.1. Canola Meal

Studies evaluating the replacement of common protein supplements by CM in dairy cow diets have consistently shown an increase in milk production. Broderick et al. [40] conducted a study where SBM was replaced by CM in isonitrogenous dairy cow diets formulated with corn and/or alfalfa silage as source of forages. The authors observed an increase of 1.0 kg/d in milk yield and 50 g/d in milk true protein, and an improvement in milk N efficiency utilization when cows were fed diets containing CM. In addition, Brito and Broderick [44] reported an increase in milk protein yield when cows were fed diets in which SBM was replaced by CM in diets containing 16.5% CP.

Two meta-analyses based on results of published peer-reviewed articles reported an increase of milk yield and components and a reduction in milk urea N (MUN) in cows fed CM compared to those fed other protein supplements [53,90]. Furthermore, Huhtanen et al. [48] conducted a meta-analysis evaluating the replacement of SBM by CM in isonitrogenous grass silage-based diets and observed an increase in the yield of milk in cows fed CM diets. Paula et al. [25] reported a significant reduction in MUN (8%) and an increase in yield of milk (1.3 kg/d), 3.5% FCM (1.2 kg/d), and ECM (0.9 kg/d) in cows fed CM compared to SBM. Brito and Broderick [44] conducted a study using similar basal diets to those used by Broderick et al. [40] and Paula et al. [25] to evaluate the performance of lactating dairy cows supplemented with isonitrogenous diets fed urea, CSM, SBM, or CM. The authors reported numerical differences in milk yield averaging 41.1, 40.5, and 40 kg/d for CM, CSM, and SBM, respectively. Yet, the authors observed a significant increase in milk protein yield for CM and SBM fed cows.

Mulrooney et al. [19] conducted a study evaluating the effects of CM replacing (100, 66, 33, and 0%) dried distillers grains with solubles (DDGS) on milk production of lactating dairy cows. They observed no differences in milk yield and components. On the other hand, by evaluating the effects of replacing CM with increased levels of wheat-DDGS (0, 10, 15, and 20% of DM), Chibisa et al. [73] reported an increase in milk yield in cows fed CM (1.8 kg/d) compared to cows fed wheat-DDGS (1.2 kg/d) In addition, the authors observed a quadratic effect for milk protein yield when wheat-DDGS was fed.

### 6.2. Camelina Meal

Camelina meal can be a valuable source of protein and energy for ruminants. It can be used to improve the fatty acid profile in milk and meat. Feeding CMM results in high concentrations of *trans*-11 18:1 and *cis*-9, *trans*-11 18:2, unaltered or slightly reduced 18:0 and *cis*-9 18:1 concentrations, and a significant reduction in total saturated fatty acids in the milk of dairy cows [75,86], sheep [93], and goats [94] as well as in sheep meat [95]. No effect on yield of milk and milk protein were found in dairy cows fed CMM [64,78]; however, there was a reduction in milk fat yield and concentration when 2 kg/d of CMM was fed to lactating cows [64]. Meanwhile, CMM had no effect on body weight [56] or ADG of growing heifers [11,58], but had a positive effect on first-service pregnancy rates, thus decreasing the cost per pregnancy [56]. Therefore, replacing various conventional protein feeds in ruminant diets with CMM has resulted in comparable milk and protein yields and ADG.

However, it has been observed that CMM may lower overall quality score (taste, flavor, texture, color, appearance, and consistency) of cheese [96], and it may also lead to changes in the physical properties of butter [64]. The change in cheese quality and butter are probably related to the effects of CMM on milk FA composition [64,75,96]. The effect of lipids on milk yield and milk fat composition are dependent on inclusion rate, degree of unsaturation, physical form, and basal diet composition [77,82,97]. The reduction in milk fat yield and concentration by CMM could be due to the depressed effects of CMM on lipogenic VFA, e.g., acetate as stated before, and the increase of *trans*-10 C18:1, which affects de novo mammary gland FA synthesis [64]. Therefore, proper CMM dietary inclusion level is highly dependent on proper basal diets.

### 6.3. Carinata Meal

To our knowledge, there are no published studies that have evaluated the effects of CRM on the performance of dairy cows.

## 7. Effects on Growth

### 7.1. Canola Meal

Studies evaluating the performance of beef cattle fed CM have been focused on replacing barley grain in the diet. A study evaluating the effects of replacing barley grain with three different types of CM (solvent-extracted CM from *B. napus*; solvent-extracted CM from *B. juncea*; and cold press-extracted CM from *B. napus*) at two levels of inclusion in the diet (15 and 30%, DM basis) on growth performance of cross bred calves on growing and finished phases did not observe differences among diets for average daily gain (ADG) in the growing and finishing periods. However, gain to feed ratio (G:F, kg/kg) was greater for solvent-extracted *B. napus* at both levels of inclusion, and solvent extracted *B. juncea* and cold press-extracted *B. napus* CM at 30% inclusion when compared to the control diet [98]. In another study evaluating the effects of replacing barley with CM, Yang et al. [99] investigated the effects of CM, wheat-DDGS, corn-DDGS, and fractionated-DDGS on growth performance of background steers and they observed an increase in final body weight, ADG, and G:F ratio for steers fed diets with CM, wheat-DDGS, and fractionated-DDGS compared to the control diet. Furthermore, Górka et al. [100] evaluated the partial replacement of barley grain and CM by high-lipid byproduct pellets on steers in the finishing phase, and they did not observe differences in final body weight and ADG; however, it a reduction in G:F ratio was observed as barley grain and CM were replaced by the high-lipid byproduct pellets. Overall, replacing barley grain with CM in finishing diets appears to be a good strategy to improve animal performance and feed efficiency of feedlot animals.

### 7.2. Camelina Meal

To our knowledge, there are no published studies that have evaluated the effects of CMM on the performance of beef cattle.

### 7.3. Carinata Meal

Average daily gain was improved when CRM was fed to beef heifers, compared to heifers fed only bermudagrass hay (0.422 and 0.141 kg/d, respectively [79]). However, when feeding CRM to dairy heifers, one study [68] reported an ADG of 0.837 kg/d, which was not different from the 0.825 kg/d obtained when dried distillers grains were fed. Frame size measurements, such as withers height, hip height, body length, heart girth, and hip width were also not affected by feeding CRM [68]. Therefore, based on a few studies [68,78,79], it can be concluded that feeding CRM can provide similar ADG compared to dried distillers grains, soybean meal, and bermudagrass hay. However, the number of studies evaluating animal performance is still very limited, and more studies evaluating these traits are warranted.

## 8. Conclusions

Overall, based on this review, we can conclude that CM is comparable and sometimes superior to SBM and other commonly used protein supplements and it can be fed to ruminants without restrictions. Studies have shown that the best animal performances (dairy cows) were obtained when CM was included at 10–16% of the diet. Camelina and carinata meals, although less investigated than CM, have been shown to have potential as valuable feedstuff for livestock animals, especially for nonlactating animals. Most studies have shown that when CMM was included at ≤10% of the diet, it did not have any detrimental effects on DMI and animal performance. However, more in vivo studies testing camelina and carinata meals in a wider range of animals, including lactating dairy animals, are warranted in order to establish safe dietary inclusion levels.

## Figures and Tables

**Table 1:** Macronutrient chemical composition of canola meal.

Item ^1^	Canola Meal	SD ^2^	*n* ^3^
Chemical composition, % of dry matter unless otherwise stated			
Dry matter, %	91.4	1.86	20
Organic matter	92.2	0.84	11
Crude protein	39.8	3.55	28
Rumen degraded protein	56.5	0.92	2
Rumen undegraded protein	43.3	1.06	2
Neutral detergent fiber	28.5	5.41	26
Acid detergent fiber	19.4	3.33	24
Ether extract	4.56	3.43	20
Ash	7.69	0.93	14
Neutral detergent insoluble nitrogen, % total N	17.5	6.52	9
Acid detergent insoluble nitrogen, % total N	5.32	1.41	8
Non-fiber carbohydrate	19.8	5.61	5
Starch	4.07	5.44	8
Lignin	9.82	1.36	4
Glucosinolates, µmol/g	5.96	2.66	4
Mineral profile, % of dry matter unless stated			
Ca	0.89	0.26	8
P	1.11	0.06	9
Mg	0.58	0.04	7
K	1.10	0.44	7
S	0.77	0.34	8
Na	0.23	0.24	5
Cl	0.11	0.03	4
Cu, mg/g	5.99	0.34	3
Fe, mg/g	179	37.3	3
Mn, mg/g	56.5	8.42	3
Mo, mg/g	1.27	0.19	3
Se, mg/g	1.11	0.01	3
Zn, mg/g	62.1	5.30	3

^1^ References [17,18,19,20,21,22,23,24,25,26,27,28,29,30,31,32,33,34,35,36,37,38,39,40,41,42,43,44,45]. ^2^ Standard deviation. ^3^ Number of studies.

**Table 2:** Micronutrient chemical composition of canola meal.

Item ^1^	Canola Meal	SD ^2^	*n* ^3^
Amino acid profile, % of total amino acid			
Histidine	2.52	0.60	6
Isoleucine	3.53	0.96	6
Leucine	6.39	1.60	6
Lysine	4.87	1.38	6
Methionine	1.88	0.57	6
Phenylalanine	3.74	0.84	6
Threonine	3.87	1.08	6
Tryptophan	1.35	0.03	3
Valine	4.47	1.23	6
Arginine	5.90	0.38	4
Total Essential AA	39.3	4.50	4
Alanine	4.43	0.15	5
Glycine	5.13	0.23	5
Proline	6.20	0.30	5
Serine	4.13	0.37	5
Tyrosine	2.90	0.20	5
Glutamic acid	22.7	8.54	4
Cysteine	2.43	0.21	4
Aspartic acid	7.34	0.31	4
Total nonessential AA	49.0	15.3	3
Vitamins, mg/kg			
Vit E	13.8	0.75	2
Pantothenic acid	9.40	0.10	2
Niacin	158	2.00	2
Choline	6600	100	2
Riboflavin	5.75	0.05	2
Biotin	1.02	0.06	2
Folic acid	1.55	0.75	2
Pyridoxine	7.10	0.10	2
Thiamin	5.15	0.05	2

^1^ References [17,18,19,20,21,22,23,24,25,26,27,28,29,30,31,32,33,34,35,36,37,38,39,40,41,42,43,44,45]. ^2^ Standard deviation. ^3^ Number of studies.

**Table 3:** Chemical composition (% of DM, unless otherwise stated) of camelina meal (CMM)

Item ^1^	Camelina Meal	SD ^2^	*n* ^3^
Chemical composition, % of dry matter unless otherwise stated			
Dry matter, %	92.2	0.99	6
Organic matter	93.9	0.49	4
Crude protein	41.9	6.19	7
Neutral detergent fiber	33.4	5.93	7
Acid detergent fiber	23.8	5.79	7
Ether extract	7.03	2.87	4
Ash	5.98	0.58	5
Glucosinolates, µmol/g	22.4	5.94	4
Mineral profile, % of dry matter unless stated			
Ca	0.31	0.03	2
P	0.82	0.13	3
Mg	0.50	-	1
K	1.50	-	1
S	1.12	-	1
Na	0.01	-	1
Cl	0.20	-	1

^1^ References [55,56,57,58,59,60,61]. ^2^ Standard deviation. ^3^ Number of studies.

**Table 4:** Amino acid composition (% of total amino acid) of camelina meal (CMM).

Item ^1^	Camelina Meal	SD ^2^	*n* ^3^
Amino acid profile, % of total amino acid			
Alanine	2.81	0.71	4
Aspartic acid	4.35	1.50	3
Cysteine	0.94	0.48	3
Glutamic acid	7.60	3.08	3
Glycine	3.00	0.88	3
Proline	2.98	0.86	3
Serine	2.81	0.78	3
Tyrosine	0.78	0.65	4
Arginine	4.13	1.51	3
Histidine	1.72	0.48	4
Isoleucine	2.17	0.68	3
Leucine	3.24	1.14	4
Lysine	2.27	0.91	4
Methionine	1.08	0.31	4
Phenylalanine	2.27	0.76	4
Threonine	1.59	0.82	4
Valine	2.81	0.94	4

^1^ References [55,57,58,59]. ^2^ Standard deviation. ^3^ Number of studies.

**Table 5:** Fatty acids composition (% of total fatty acids) of camelina meal (CMM).

Item ^1^	Camelina Meal	SD ^2^	*n* ^3^
Fatty acid composition, % of total ^1^			
C16:0	8.21	1.22	4
C18:0	2.51	0.27	3
C18:1	17.9	2.73	4
C18:2n-6	25.4	2.99	4
C18:3n-3	29.1	7.13	4
C20:0	0.81	0.84	1
C22:1 n-9	2.38	1.11	3

^1^ References [56,58,64,65]. ^2^ Standard deviation. ^3^ Number of studies.

**Table 6:** *Brassica carinata* seed fatty acid profile.

Fatty Acid Composition, % of Total ^1^	*B. carinata*	*B. carinata* (Low Erucic Acid)
C16:0	4 to 6	5.5
C18:0	1.3	0.5
C18:1	10 to 17	42 to 44
*cis*-9, *cis*-12, C18:2	17 to 25	35 to 37
*cis*-9, *cis*-12, *cis*-15, C18:3	10 to 17	15 to 16
C20:0	0.7	-
C22:1	45.4	-
Total fatty acid	42	-

^1^ References [13,61,66].

**Table 7:** Chemical composition of *Brassica carinata* (% of DM, unless otherwise stated).

Item	*B. carinata*			
	Seed ^1^	Cold Pressed ^2^	Pressed Cake ^3^	Meal ^4^
Crude protein	24.8 to 23.1	38.7	48.5	48.17 to 53
Neutral detergent fiber	9.67 to 13.4	20	10.2	14. to 18.8
Acid detergent fiber	6.43 to 4.95	12	6.7	10.2 to 11.4
Ether extract	38.5 to 40.4	20.1	2.5	0.3
Glucosinolates, µmol/g	-	43.97	168.5	115.2
Rumen undegraded protein, % crude protein	-	-	10.7	23.7
Rumen degraded protein, % of crude protein	-	-	89.3	76.3

^1^ [11,69]; ^2^ [14]; ^3^ [68]; ^1–4^ = oil extraction method.
